# The deficiency of methylglyoxal synthase promotes cell proliferation in *Synechocystis* sp. PCC 6803 under mixotrophic conditions

**DOI:** 10.5511/plantbiotechnology.24.0718a

**Published:** 2024-12-25

**Authors:** Aikeranmu Kadeer, Yuuma Ishikawa, Kaushalya Dayarathne, Atsuko Miyagi, Toshiki Ishikawa, Masatoshi Yamaguchi, Maki Kawai-Yamada

**Affiliations:** 1 Graduate School of Science and Engineering, Saitama University, 255 Shimo-Okubo, Sakura-ku, Saitama-city, Saitama 338-8570, Japan; 2 Institute for Molecular Physiology and Cluster of Excellence on Plant Sciences (CEPLAS), Heinrich-Heine-University Düsseldorf, Universitätsstraße 40225, Germany; 3 Faculty of Agriculture, Yamagata University, 1-23 Wakabacho, Tsuruoka City, Yamagata 997-8555, Japan

**Keywords:** cyanobacteria, gene disruption, methylglyoxal, methylglyoxal synthase

## Abstract

Methylglyoxal synthase (MGS), which converts dihydroxyacetone phosphate to methylglyoxal (MG), is found in only prokaryotes. *Synechocystis* sp. PCC 6803 possesses the gene *sll0036*, which encodes MGS. To clarify the biological function of MGS, we constructed a gene-disruption strain of *Synechocystis* sp. PCC 6803. Expression analysis showed that MG metabolic genes (*sll0036*, *sll0067*, and *slr1167*) were upregulated under photoautotrophic conditions compared to mixotrophic conditions. The *sll0036*-deficient strain (Δ0036) exhibited a higher growth rate than the wild-type (WT) strain under mixotrophic conditions, whereas no significant difference was observed under photoautotrophic conditions. When cells were cultured in a medium supplemented with sorbitol or mannitol instead of glucose, the growth enhancement observed in the Δ0036 strain disappeared. This suggests that the difference in growth between Δ0036 and WT is influenced by glucose-related metabolism rather than osmotic stress. MG contents were found to be decreased in the Δ0036 strain compared to WT under mixotrophic conditions. This suggests that the reduction of MG level might activate the cell proliferation of *Synechocystis* sp. PCC 6803 under mixotrophic conditions.

## Introduction

Methylglyoxal (MG), a highly reactive cytotoxic alpha-oxoaldehyde compound found in all organisms, from prokaryotes to eukaryotes, is produced as a byproduct of various metabolic reactions in cells (Cooper 1984). MG is synthesized by enzymatic or nonenzymatic reactions, and it is particularly upregulated under stress conditions (Thornalley 1996). MG is non-enzymatically produced from lipids and glucose catabolism. Non-enzymatic decarboxylation and oxidation of acetoacetate (a derivative of lipid catabolism), and degradation of glycolytic intermediates such as dihydroxyacetone phosphate (DHAP) and glyceraldehyde 3-phosphate lead to MG production (Kold-Christensen and Johannsen 2020; Li 2016). In the enzymatic reaction, MG synthase (MGS) converts DHAP to MG (Yuan and Gracy 1977). MG is primarily eliminated by MG reductase and through the glyoxalase pathway, where it conjugates with glutathione (GSH) to form non-toxic lactic acid (Mocchetti et al. 2022). Glutathione *S*-transferase (GST) catalyzes the conjugation of GSH with MG, the first step of MG detoxification (Kammerscheit et al. 2020).

Elevated concentrations of MG can lead to cell damage and death by reacting with DNA, RNA, and proteins (Jain et al. 2018). In *Escherichia coli*, MG inhibits cell growth by interfering with protein synthesis and consequently preventing the initiation of DNA replication (Fraval and McBrien 1980).

Conversely, MG has been reported to be required as a signaling molecule in bacteria (Campbell et al. 2007) and plants (Hoque et al. 2016). In human cells, MG induces retinal pigment epithelial cell death through a caspase-independent manner, which relies on reactive oxygen species (ROS) formation, mitochondrial membrane potential loss, intracellular calcium elevation, and endoplasmic reticulum stress response (Chan et al. 2016). In Arabidopsis, MG induces stomatal closure accompanied by intracellular ROS accumulation and cytosolic calcium ion oscillation (Hoque et al. 2012). Therefore, the amount of MG within cells might be strictly controlled through the involvement of MGS or MG degradation pathway.

*E. coli* has a single *MGS* gene (*mgsA*) (Tötemeyer et al. 1998). In a previous study, a mutant lacking *MGS* was constructed, resulting in the co-metabolism of glucose and xylose, and accelerated sugar utilization. However, it did not show any difference in cell proliferation compared to WT strains on media containing a range of carbon sources (Tötemeyer et al. 1998; Yomano et al. 2009). Therefore, MGS is thought to limit sugar utilization in *E. coli*. A study reported a significant metabolic change in cyanobacteria *Synechococcus elongatus* PCC 7942 that enabled the production of 1,2-propanediol from DHAP by introducing *E. coli mgsA* along with several metabolic genes (Li and Liao 2013). Therefore, understanding the role of MG and MGS is an interesting area of research that may have implications for biotechnological applications, including the development of novel industrial products.

*Synechocystis* sp. PCC 6803 is a unicellular freshwater cyanobacterial strain and one of the widely used model organisms for studying various cellular processes, including photosynthesis, carbon and nitrogen assimilation, and adaptations to environmental stresses. Unlike the non-photosynthetic organism *E. coli*, the photosynthetic bacterium *Synechocystis* sp. PCC 6803 can grow in photoautotrophic culture, which supplies carbon through photosynthesis, as well as in mixotrophic culture, which supplies carbon through both photosynthesis and sugars in the medium.

In *Synechocystis* sp. PCC 6803, MGS is encoded by the *sll0036* gene. To clarify the functions of MGS in cyanobacteria, we constructed a *sll0036*-deficient mutant (Δ0036) of *Synechocystis* sp. PCC 6803 and analyzed the effect for photoautotrophic or mixotrophic growth. As a result, we demonstrated the physiological roles of *sll0036* as a cell growth suppressor under mixotrophic conditions.

## Materials and methods

### Strains and culture conditions

*Synechocystis* sp. PCC 6803 was used as the wild-type strain (WT). For the photoautotrophic culture conditions, strains were cultured in liquid BG-11 medium (Rippka et al. 1979) on a shaker (100 rpm) at 30°C under continuous light at 30 µE m^−2^ s^−1^. Cell growth was monitored by optical density at 730 nm (OD_730_) measured using a spectrophotometer (Ultrospec 3000; Pharmacia Biotech, Midland, Canada). For the mixotrophic conditions, 5 mM glucose (sterilized by filtration) was added to the BG-11 medium and cultured on a shaker (100 rpm) at 30°C under continuous light at 30 µE m^−2^ s^−1^. To obtain the *sll0036*-deficient mutant (Δ0036), the *sll0036* gene was replaced with the spectinomycin resistance gene by homologous recombination. After the selection of spectinomycin (50 µg ml^−1^) resistant colonies, segregation was checked by PCR with the following primers: Δ0036: forward, 5′-ATGGCTGCCCATATAGCCCTTATCTCCCAC-3′, reverse, 5′-TCAACTGTTTTCCACTGGAGCAAAGATGTTT-3′. Amplified PCR products were electrophoresed in 1% agarose gel.

### Expression analysis

WT cells were cultured in liquid culture (20 ml, OD_730_=0.01) under photoautotrophic or mixotrophic conditions for 4 days. Cells were collected by centrifugation (4,000×g, 10 min), and the cell pellet was ground to a powder under freezing conditions using liquid nitrogen. RNA was extracted using the guanidinium thiocyanate-phenol-chloroform method with some modifications (Chomczynski and Sacchi 1987). The extracted total RNA was further purified using a RNeasy Plant Mini Kit (Qiagen, Venlo, The Netherlands) and RNase-Free DNase set (QIAGEN, Hilden, Germany) according to the manufacturer’s instructions. Using a high-capacity cDNA reverse transcription kit (Thermo Fisher Scientific, MA, USA), cDNA was synthesized according to the manufacturer’s instructions. Amplification of *sll0036* gene transcripts was performed using Quick taq HS dye mix (TOYOBO, Osaka, Japan) and a thermal cycler (2720; Applied Biosystems, Tokyo, Japan). Quantitative RT-PCR (qRT-PCR) analysis was performed using Power SYBR Green PCR Master Mix (ABI Prism®) and the 7300 Real-Time PCR system (Applied Biosystems) with the following primers; *sll0036*: forward, 5′-ATGGCTGCCCATATAGCCCTTATCTCCCAC-3′; reverse, 5′-TCAACTGTTTTCCACTGGAGCAAAGATGTT-3′, *sll0067*: forward, 5′-ATGATCAAACTATACGGTGCCCCCC-3′; reverse, 5′-TCAGCGGGCACCGATGGAAGCTTGG-3′, *slr1167*: forward, 5′-ATGGCCCCATCAATTTCCCCTGTC-3′; reverse, TTAAAGACCGGAATTATCTTGAAT-3′, *rnPB*: forward, 5′-GCAAAGGTGCGGTAAGAG-3′; reverse, 5′-GGGGCAGGAAAAAGACCA-3′.

### Growth comparison

For the photometric growth analysis, 4-day-old cells were diluted (OD_730_=0.01) with a new medium and cultured under photoautotrophic or mixotrophic conditions. The optical density at 730 nm (OD_730_) of each culture was measured at 1 day intervals. To investigate the effect of carbon source on the growth, BG-11 medium supplied with 5 mM sorbitol or 5 mM mannitol was used.

### MG measurement

MG measurement was performed according to the protocol described by Jain et al. (2018) with modifications. The WT and Δ0036 strains were cultured under photoautotrophic or mixotrophic conditions for 4 days prior to the measurement, and the cells were collected by centrifugation (3,500×g). Subsequently, the cell pellet was resuspended in 250 µl of 5 M perchloric acid to prepare a cell lysate. After incubation on ice for 15 min, the lysate was centrifuged at 12,500×g for 10 min. The resulting supernatant was neutralized using 1 M Na_2_HPO_4_, followed by a second centrifugation step. The resulting supernatant was transferred to a new tube, and 10 µl of 100 mM NaN_3_ was added per 1 ml of the supernatant. A reaction mixture containing 250 µl of 7.2 mM 1,2-diaminobenzene, 100 µl of 5 M perchloric acid, and 650 µl of the final supernatant was prepared. The reaction mixture was incubated in darkness at 25°C for 3 h, and absorbance was measured at 336 nm by spectrophotometer. The absorbance of the reaction mixture measured at 336 nm before the incubation was considered the blank reading. MG contents of WT and Δ0036 strains were calculated using a standard curve.

## Results

MG is produced enzymatically as a byproduct of glycolysis or nonenzymatically through the catabolism of lipids and glucose in *Synechocystis* sp. PCC 6803. The enzyme MGS, encoded by the gene *sll0036* (Kammerscheit et al. 2020) facilitates the conversion of DHAP to MG (Supplementary Figure S1). The MGS of *Synechocystis* sp. PCC 6803 has a unique structure possessing two functional domains: the MGS motif and the NAD kinase (NADK) motif (Supplementary Figures S2, S3). The MGS motif is a region that is highly common among various MGS proteins, but its structure and function remain poorly understood. On the other hand, the NADK motif is a functional domain conserved in NAD kinase, which phosphorylates NAD(H) to NADP(H) (Ishikawa and Kawai-Yamada 2019). In previous reports, only procaryotes were known to possess the *MGS* gene (Tötemeyer et al. 1998). Interestingly, as a result of the database search, only certain species of cyanobacteria had MGS that includes both the MGS motif and NADK motif, while the NADK motif was absent in the MGS of other species. However, since the ATP-binding region of the NADK motif is not conserved in Sll0036, the Sll0036 is unlikely to have kinase activity.

**Figure 1:**
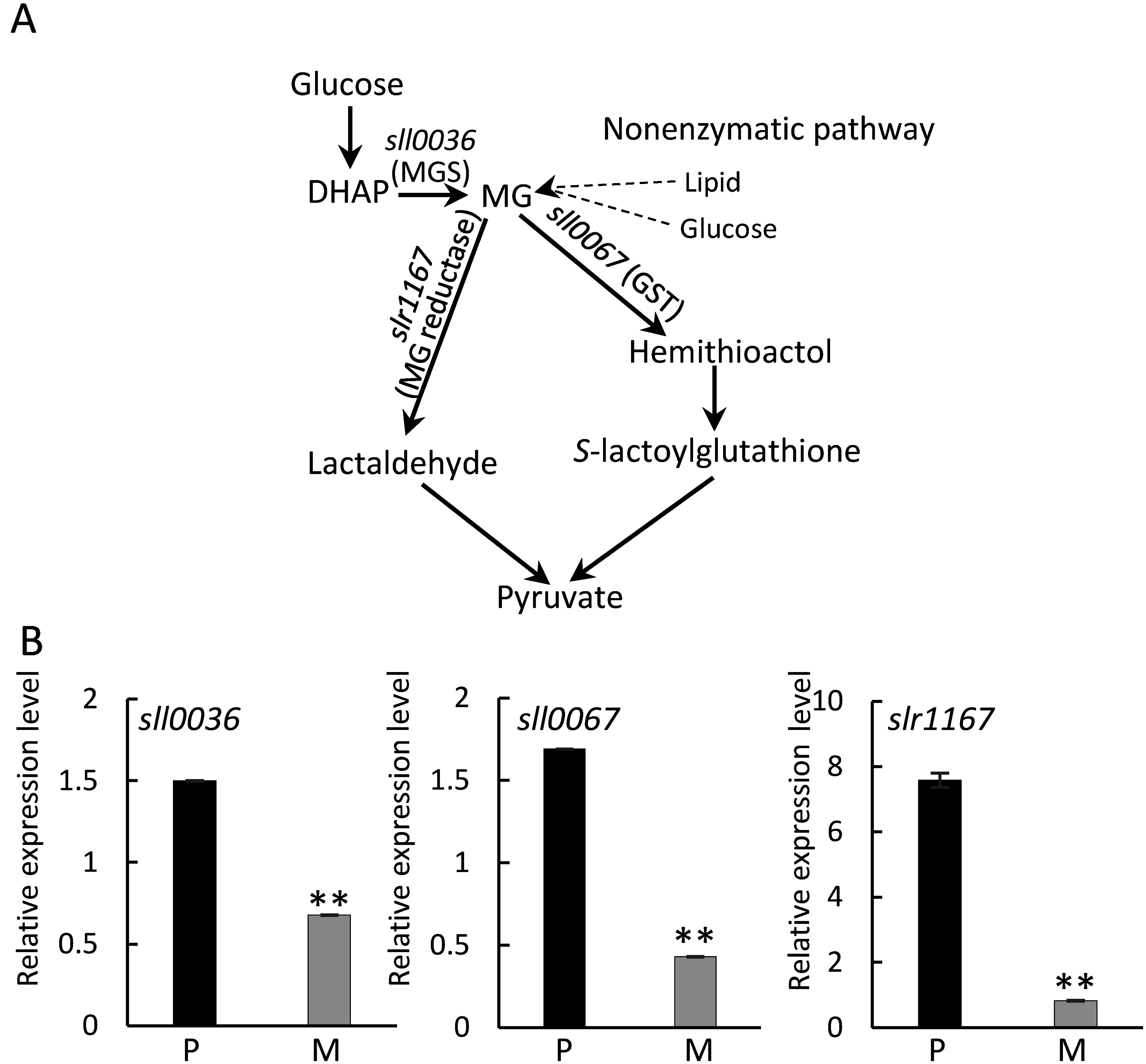
Figure 1. Expression analysis of MG-related genes in *Synechocystis* sp. PCC 6803. (A) Synthesis and decomposition of MG. The dotted lines indicate non-enzymatic synthesis. Gene expression of *sll0036* encoding MGS, *sll0067* encoding GST, and *slr1167* encoding MG reductase was assessed. (B) qPCR of MG-related genes (*sll0036*, *sll0067*, and *slr1167*). RNA was purified from cells cultured under photoautotrophic (P) or mixotrophic (M) conditions for 4 days. Expression levels are represented relative to the internal control (*16S rRNA*). *n*=3, values are mean±SD, ** *p*<0.01.

We investigated the expression of the MGS encoding gene *sll0036* and MG degradation-related genes, *sll0067*, which encodes glutathione *S*-transferase (GST) and *slr1167*, which encodes MG reductase (Figure 1A, B). The results of qRT-PCR showed a higher expression level of MG-related genes in photoautotrophic compared to mixotrophic conditions (Figure 1B).

**Figure 2:**
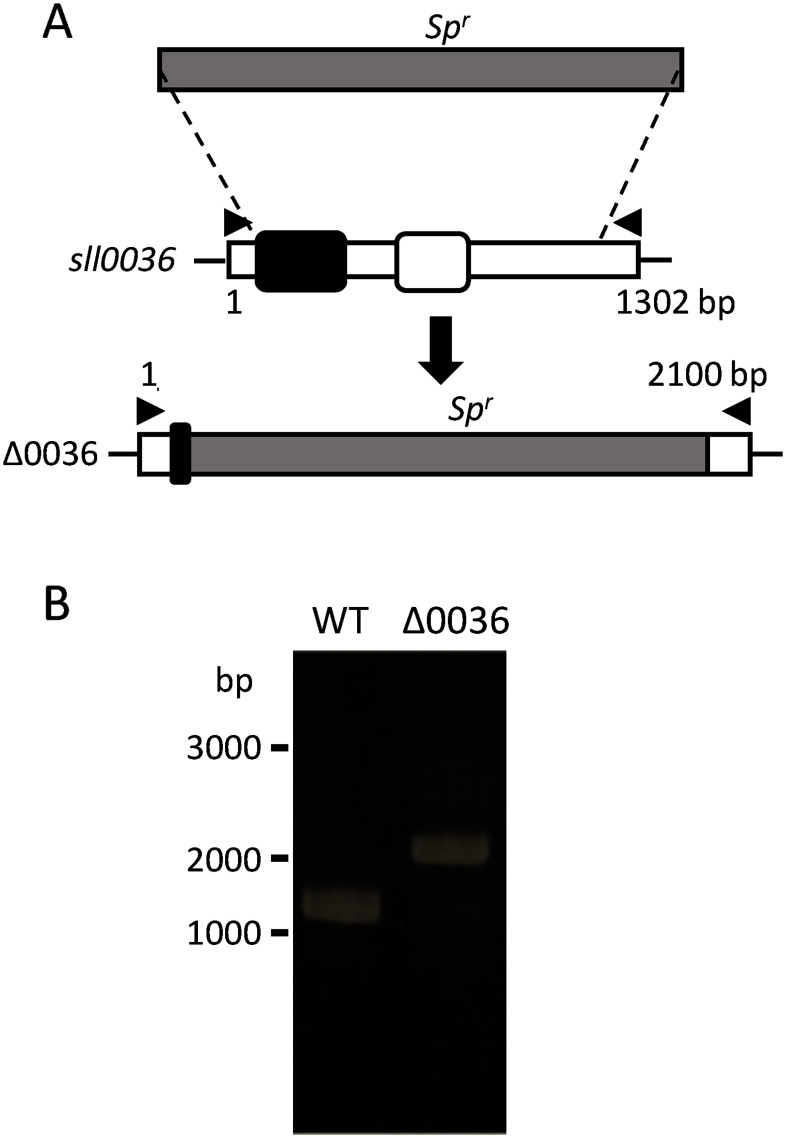
Figure 2. Schematic representation of the construction and segregation check of the Δ0036 strain of *Synechocystis* sp. PCC 6803. (A) The *sll0036* gene was replaced with the spectinomycin resistance gene (*Sp^r^*). Arrowheads show the location of primers used for segregation tests (B). Black box, MG synthase motif; white box, NADK motif. (B) Segregation check of the mutant strain Δ0036. PCR products amplified with primers shown in (A) were separated by agarose gel electrophoresis.

**Figure 3:**
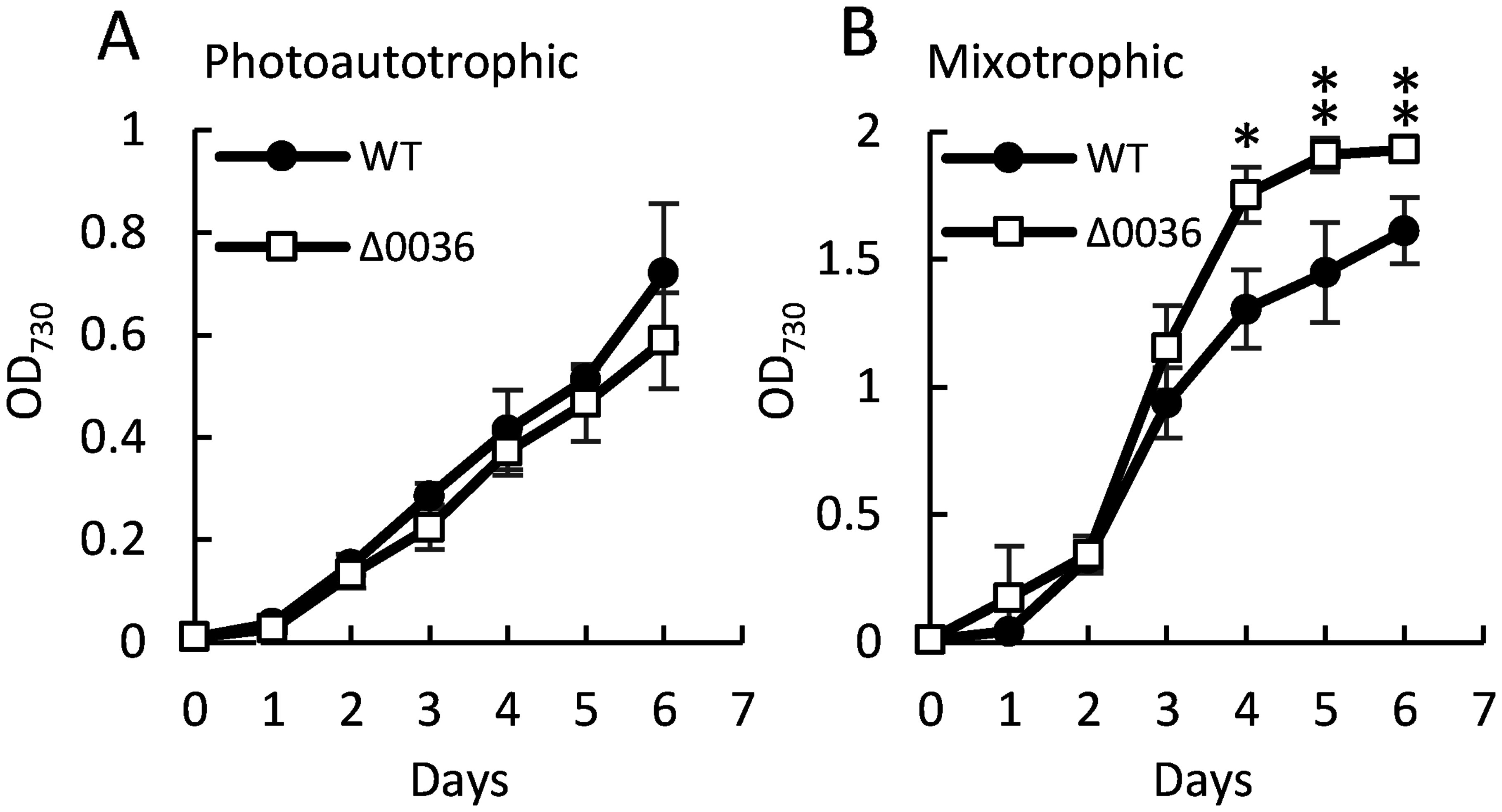
Figure 3. Growth comparison of Δ0036 and WT cultured under photoautotrophic (A) and mixotrophic (B) conditions. OD_730_ was measured at each time point. *n*=3, values are mean±SD, * *p*<0.05, ** *p*<0.001.

**Figure 4:**
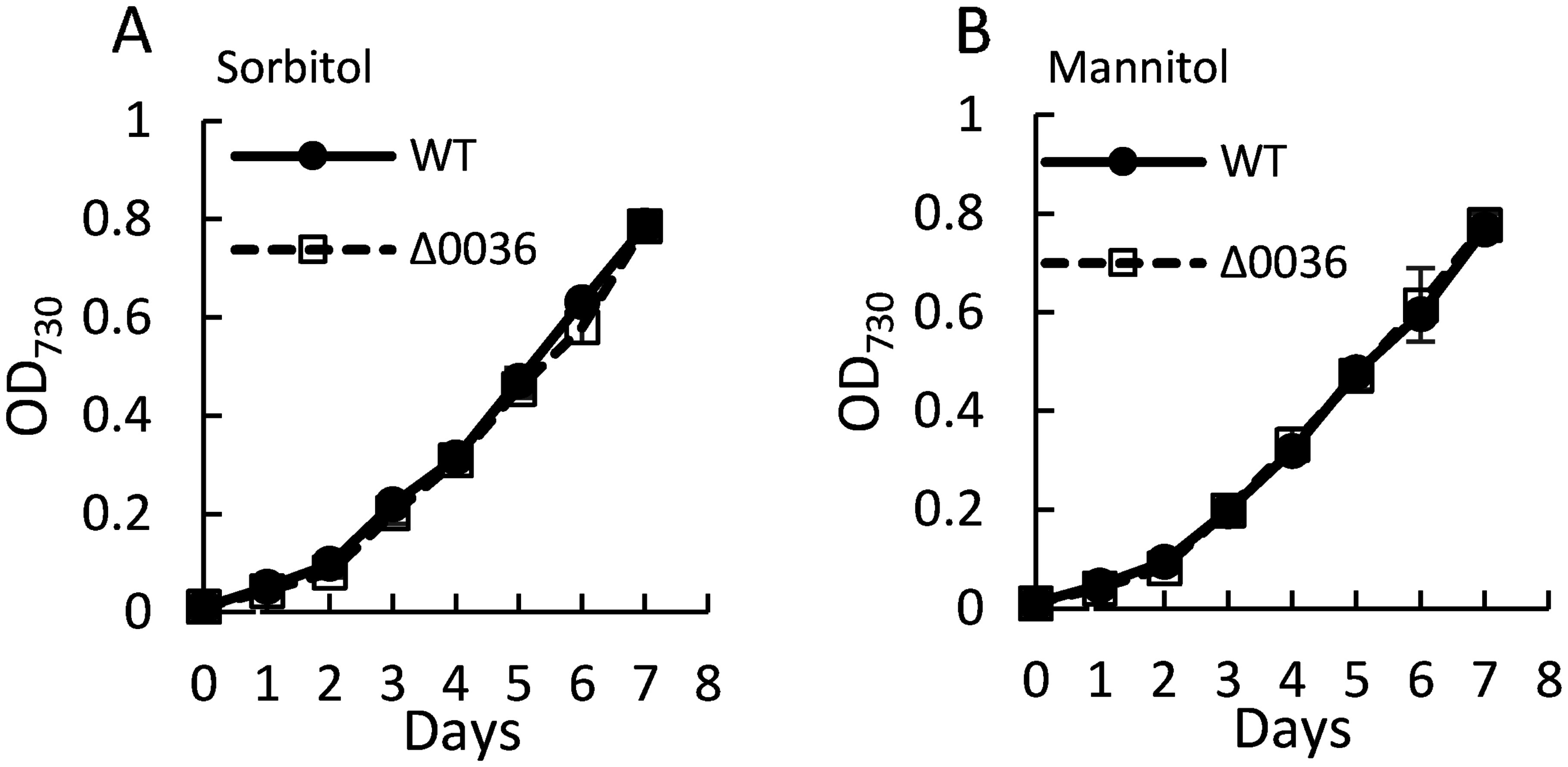
Figure 4. Growth comparison of Δ0036 and WT cultured in BG-11 medium with 5 mM sorbitol (A) and with 5 mM mannitol (B). OD_730_ was measured at each time point. *n*=3, values are mean±SD, *p*>0.05.

To investigate the biological function of MG in *Synechocystis* sp. PCC 6803, we analyzed the Δ0036 strain, in which the *sll0036* gene was replaced with a spectinomycin resistance cassette (Figure 2A). Figure 2B shows the results of genomic PCR confirming the replacement of *sll0036* with the spectinomycin resistance gene and complete segregation. The growth of Δ0036 and WT strains were compared under photoautotrophic and mixotrophic conditions. Under photoautotrophic conditions, both WT and Δ0036 strains exhibited similar growth rates, with no significant differences observed (Figure 3A). However, under mixotrophic conditions, Δ0036 showed a higher growth rate than the WT strain (Figure 3B). To examine whether the growth promotion of Δ0036 under mixotrophic conditions depends on the carbon source of the medium, growth comparison was performed in BG-11 medium supplemented with sorbitol or mannitol. As shown in Figure 4, no growth promotion effect in Δ0036 was observed in the media supplemented with sorbitol or mannitol. This result indicates that glucose in the medium is responsible for the phenotype of Δ0036.

**Figure 5:**
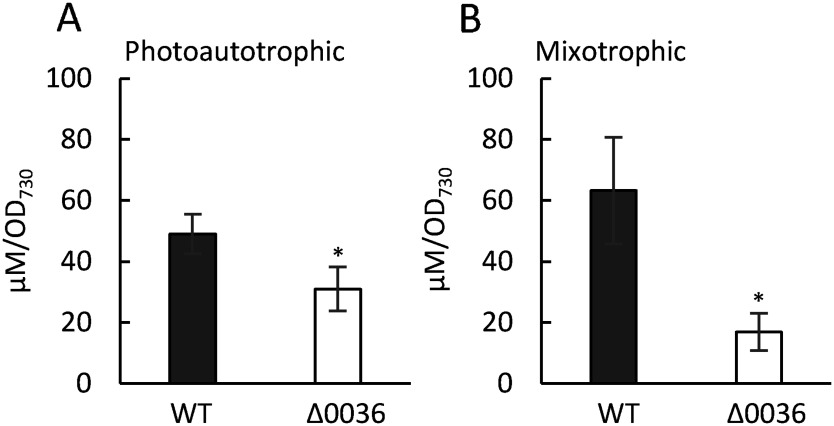
Figure 5. Measurement of MG in Δ0036 strains. Cells cultured for 4 days under photoautotrophic (A) and mixotrophic (B) conditions were used for the MG measurements. Values are mean±SD of three independent experiments, * *p*<0.05.

To investigate whether the amount of MG within cells is related to differences in cell proliferation, MG was quantified using the photometric method. The Δ0036 strain exhibited lower MG contents compared to the WT strain under both photoautotrophic and mixotrophic conditions (Figure 5A, B). These results indicate that the disruption of *sll0036* contributed to the decreased MG contents within cells and enhancement in cell growth only under mixotrophic conditions.

## Discussion

In this study, we aimed to analyze the biological function of *sll0036* encoding MGS in *Synechocystis* sp. PCC 6803. Expression analysis showed *MGS* (*sll0036*) and MG-degradation-related genes (*sll0067* and *slr1167*) were upregulated under photoautotrophic condition compared to mixotrophic condition (Figure 1). The higher expression of MG-related genes under photoautotrophic condition might be due to the higher MG production induced by transportation of sugars synthesized through photosynthesis to the MG pathway. WT strain showed a decrease in the expression of MG-related genes (Figure 1), but an increase in MG contents (Figure 5) under mixotrophic conditions. Since MG also can be produced non-enzymatically from intermediates of glucose catabolism as shown in Figure 1A, we suspect that the excess glucose involved in the medium may activate non-enzymatic MG synthesis in the WT strain under mixotrophic conditions. The MGS deficiency in the Δ0036 strain resulted in reduced MG contents compared to WT under both photoautotrophic and mixotrophic culture conditions (Figure 5). The higher growth rate observed in the Δ0036 strain under mixotrophic conditions, along with no change in growth rate under photoautotrophic conditions compared to the WT, suggest that a decrease in the amount of MG may be associated with growth promotion only under mixotrophic conditions.

The contribution of MGS to nutrient metabolism was reported in *E. coli*. The deletion of MGS in *E. coli* reduced the MG generation and resulted in accelerated glucose catabolism, although it did not show a difference in growth (Yomano et al. 2009). Since the MGS deletion mutant releases the restriction on sugar utilization of *E. coli*, it is thought to play a role in suppressing sugar metabolism (Grabar et al. 2006; Tötemeyer et al. 1998; Yomano et al. 2009; Zhu et al. 2001). In our present data that the Δ0036 strain showing a phenotype only under mixotrophic conditions may be related to the fact that MGS is thought to be a repressor of sugar metabolism in *E. coli*, however, the difference is that Δ0036 showed accelerated growth to WT under mixotrophic conditions (Figure 3).

*Synechocystis* sp. PCC 6803 prefers mixotrophic culture conditions and shows faster growth compared to the photoautotrophic condition (Takahashi et al. 2008). Under the mixotrophic condition, cells can use both carbon sources from photosynthesis and glucose from the medium (Yoshikawa et al. 2013). In addition, MG is estimated to inhibit glucose metabolism in *E. coli* (Tötemeyer et al. 1998; Yomano et al. 2009). These may be the reasons for the accelerated growth of Δ0036 only under the mixotroph. However, the details of the mechanism remain unclear and further research is needed.

DHAP is converted to MG, whereby orthophosphate is liberated which can be used in glycolysis by the glyceraldehyde-3-phosphate dehydrogenase (GAPDH) for the conversion of GAP to 1,3-bisphosphoglycerate (David et al. 2019). The produced MG is converted to lactate and finally to pyruvate without further production of ATP (Cooper 1984). The activity of the MG pathway is also part of the overflow metabolism when an excess of carbohydrates enters central metabolism (Tötemeyer et al. 1998). In animals, GAPDH is one of the targets of MG-induced cytotoxicity (Barinova et al. 2019; Lee et al. 2005; Muronetz et al. 2019). It is possible to speculate that GAPDH may also play a key role in the regulation of MG-related proliferation in cyanobacteria. According to applied an integrated transcriptomic and metabolomic analysis revealed that the oxidative pentose phosphate and glycolysis pathways of *Synechocystis* 6803 were activated under mixotrophic conditions with glucose rather than autotrophic conditions (Yoshikawa et al. 2013). Furthermore, previous research mentions that the addition of glucose can dramatically promote biomass accumulation in the model cyanobacterium *Synechocystis* sp. PCC 6803 (Wang et al. 2002). Since the Δ0036 strain mostly depends on the non-enzymatic pathway to synthesize MG, its glucose utilization for MG synthesis is lower compared to the WT strain which uses more glucose for MG synthesis via both enzymatic and non-enzymatic pathways. Therefore, it can be speculated that the Δ0036 strain has more available glucose for energy production, which is required for cell proliferation, resulting in higher growth under mixotrophic conditions compared to the WT strain.

MGS is found only in prokaryotes, and Sll0036 has both the MGS motif and the NADK motif (Supplementary Figure S3). The enzyme NADK synthesizes NADP^+^ from NAD^+^ and ATP. *Synechocystis* sp. PCC 6803 has two NADKs (Sll1415 and Slr0400), and they have crucial roles in the regulation of cyanobacterial metabolism by controlling of the NADP^+^/NAD^+^ ratio (Ishikawa and Kawai-Yamada 2019; Ishikawa et al. 2016, 2019, 2021). NADK motif includes the NAD^+^-binding site (GGDG) and ATP-binding site (NE/ND), and both Sll1415 and Slr0400 have typical NADK motifs (Gao and Xu 2012). However, since Sll0036 has only the NAD^+^-binding site, it is not expected to have NADK activity. It is known that the balance of NAD(P)(H) is altered depending on the metabolic status of cells, which in turn controls metabolic enzyme activities (Anderson et al. 2017). The MGS activity of Sll0036 may also be controlled through the NAD^+^-binding region. Biochemical research on the regulation of enzymatic activity of MGS is required in the future.

*Synechocystis* sp. PCC 6803 is a model organism used in research on material production through metabolic engineering (Angermayr et al. 2014). In this study, we revealed that cell proliferation of the Δ0036 strain was promoted under mixotrophic conditions. Deletion of the *sll0036* gene results in the disruption of the bypass pathway for carbon metabolism through MG. In the WT strain, carbon can flow through both the glycolysis and MG pathways, whereas in Δ0036, carbon can only flow through the glycolysis. Under photoautotrophic conditions, carbon sources only come from photosynthesis. However, in mixotrophic conditions, the medium contains extra glucose (Takahashi et al. 2008; Yoshikawa et al. 2013), which might allow more external carbon to be used for cell proliferation. In contrast, the WT strain might use MG metabolism to suppress the use of excess sugar and control cell proliferation.

In conclusion, MGS has a significant role in controlling cyanobacterial growth. The deficiency of MGS promotes cell proliferation under mixotrophic conditions, likely due to an increase in available glucose required for energy production. Given the importance of enhanced cell growth for higher productivity of cyanobacterial metabolites with industrial importance, the higher cell proliferation shown by the Δ0036 strain under mixotrophic conditions, as described in this study, can be considered for improving the growth of industrially important cyanobacterial in future applications.

## Abbreviations

DHAP: dihydroxyacetone phosphate
GAPDH: glyceraldehyde-3-phosphate dehydrogenase
GSH: glutathione
GST: glutathione *S*-transferases
MG: methylglyoxal
MGS: methylglyoxal synthase
NAD^+^: nicotinamide adenine dinucleotide
NADK: NAD kinase

## Description of Supplementary Files

Supplementary Figure S1. Primary metabolism and MG synthesis pathway of cyanobacteria.

Supplementary Figure S2. Comparison of genes showing homology with the NADK motif of cyanobacteria.

Supplementary Figure S3. Molecular phylogenetic tree of genes with MG synthase motifs.

## References

[RAnderson2017] Anderson KA, Madsen AS, Olsen CA, Hirschey MD (2017) Metabolic control by sirtuins and other enzymes that sense NAD^+^, NADH or their ratio. *Biochim Biophys Acta Bioenerg* 1858: 991–998 28947253 10.1016/j.bbabio.2017.09.005 PMC5648639

[RAngermayr2014] Angermayr SA, Van der Woude AD, Correddu D, Vreugdenhil A, Verrone V, Hellingwerf KJ (2014) Exploring metabolic engineering design principles for the photosynthetic production of lactic acid by *Synechocystis* sp. PCC 6803. *Biotechnol Biofuels* 7: 99 24991233 10.1186/1754-6834-7-99 PMC4078008

[RBarinova2019] Barinova K, Serebryakova M, Sheval E, Schmalhausen E, Muronetz V (2019) Modification by glyceraldehyde-3-phosphate prevents amyloid transformation of alpha-synuclein. *Biochim Biophys Acta Proteins Proteom* 1867: 396–404 30639428 10.1016/j.bbapap.2019.01.003

[RCampbell2007] Campbell AK, Naseem R, Holland IB, Matthews SB, Wann KT (2007) Methylglyoxal and other carbohydrate metabolites induce lanthanum-sensitive Ca^2+^ transients and inhibit growth in *E. coli.* *Arch Biochem Biophys* 468: 107–113 17961498 10.1016/j.abb.2007.09.006

[RChan2016] Chan CM, Huang DY, Huang YP, Hsu SH, Kang LY, Shen CM, Lin WW (2016) Methylglyoxal induces cell death through endoplasmic reticulum stress-associated ROS production and mitochondrial dysfunction. *J Cell Mol Med* 20: 1749–1760 27307396 10.1111/jcmm.12893 PMC4988286

[RChomczynski1987] Chomczynski P, Sacchi N (1987) Single-step method of RNA isolation by acid guanidinium thiocyanate-phenol-chloroform extraction. *Anal Biochem* 162: 156–159 2440339 10.1006/abio.1987.9999

[RCooper1984] Cooper RA (1984) Methylglyoxal in microorganisms. *Annu Rev Microbiol* 38: 49–68 6093685 10.1146/annurev.mi.38.100184.000405

[RDavid2019] David C, Schmid A, Bühler K (2019) Cellular physiology controls photoautotrophic production of 1,2-propanediol from pools of CO_2_ and glycogen. *Biotechnol Bioeng* 116: 882–892 30480779 10.1002/bit.26883

[RFraval1980] Fraval HNA, McBrien DCH (1980) The effect of methylglyoxal on cell division and the synthesis of protein and DNA in synchronous and asynchronous cultures of *Escherichia coli* B/r. *J Gen Microbiol* 117: 127–134 6993622 10.1099/00221287-117-1-127

[RGao2012] Gao H, Xu X (2012) The cyanobacterial NAD kinase gene *sll1415* is required for photoheterotrophic growth and cellular redox homeostasis in *Synechocystis* sp. strain PCC 6803. *J Bacteriol* 194: 218–224 22056937 10.1128/JB.05873-11 PMC3256667

[RGrabar2006] Grabar TB, Zhou S, Shanmugam KT, Yomano LP, Ingram LO (2006) Methylglyoxal bypass identified as source of chiral contamination in L(+) and D(−)-lactate fermentation by recombinant *Escherichia coli.* *Biotechnol Lett* 28: 1527–1535 16868860 10.1007/s10529-006-9122-7

[RHoque2016] Hoque TS, Hossain MA, Mostofa MG, Burritt DJ, Fujita M, Tran LSP (2016) Methylglyoxal: An emerging signaling molecule in plant abiotic stress responses and tolerance. *Front Plant Sci* 7: 1341 27679640 10.3389/fpls.2016.01341 PMC5020096

[RHoque2012] Hoque TS, Uraji M, Ye W, Hossain MA, Nakamura Y, Murata Y (2012) Methylglyoxal-induced stomatal closure accompanied by peroxidase-mediated ROS production in Arabidopsis. *J Plant Physiol* 169: 979–986 22437147 10.1016/j.jplph.2012.02.007

[RIshikawa2021] Ishikawa Y, Cassan C, Kadeer A, Yuasa K, Sato N, Sonoike K, Kaneko Y, Miyagi A, Takahashi H, Ishikawa T, et al. (2021) The NAD kinase Slr0400 functions as a growth repressor in *Synechocystis* sp. PCC 6803. *Plant Cell Physiol* 64: 668–677 10.1093/pcp/pcab023 33560438

[RIshikawa2019a] Ishikawa Y, Kawai-Yamada M (2019) Physiological significance of NAD kinases in cyanobacteria. *Front Plant Sci* 10: 847 31316540 10.3389/fpls.2019.00847 PMC6610520

[RIshikawa2019b] Ishikawa Y, Miyagi A, Haishima Y, Ishikawa T, Nagano M, Yamaguchi M, Hihara Y, Kaneko Y, Kawai-Yamada M (2019) One of the NAD kinases, *sll1415*, is required for the glucose metabolism of *Synechocystis* sp. PCC 6803. *Plant J* 98: 654–666 30693583 10.1111/tpj.14262

[RIshikawa2016] Ishikawa Y, Miyagi A, Haishima Y, Ishikawa T, Nagano M, Yamaguchi M, Hihara Y, Kawai-Yamada M (2016) Metabolomic analysis of NAD kinase-deficient mutants of the cyanobacterium *Synechocystis* sp. PCC 6803. *J Plant Physiol* 205: 105–112 27657983 10.1016/j.jplph.2016.09.002

[RJain2018] Jain M, Nagar P, Sharma A, Batth R, Aggarwal S, Kumari S, Mustafiz A (2018) GLYI and D-LDH play key role in methylglyoxal detoxification and abiotic stress tolerance. *Sci Rep* 8: 5451 29615695 10.1038/s41598-018-23806-4 PMC5883029

[RKammerscheit2020] Kammerscheit X, Hecker A, Rouhier N, Chauvat F, Cassier-Chauvat C (2020) Methylglyoxal detoxification revisited: Role of glutathione transferase in model cyanobacterium *Synechocystis* sp. strain PCC 6803. *MBio* 11: e00882-20 32753490 10.1128/mBio.00882-20 PMC7407080

[RKold2020] Kold-Christensen R, Johannsen M (2020) Methylglyoxal metabolism and aging-related disease: Moving from correlation toward causation. *Trends Endocrinol Metab* 31: 81–92 31757593 10.1016/j.tem.2019.10.003

[RLee2005] Lee HJ, Howell SK, Sanford RJ, Beisswenger PJ (2005) Methylglyoxal can modify GAPDH activity and structure. *Ann N Y Acad Sci* 1043: 135–145 16037232 10.1196/annals.1333.017

[RLi2013] Li H, Liao J (2013) Engineering a cyanobacterium as the catalyst for the photosynthetic conversion of CO_2_ to 1,2-propanediol. *Microb Cell Fact* 12: 4 23339487 10.1186/1475-2859-12-4 PMC3556108

[RLi2016] Li ZG (2016) Methylglyoxal and glyoxalase system in plants: Old players, new concepts. *Bot Rev* 82: 183–203

[RMocchetti2022] Mocchetti E, Morette L, Mulliert G, Mathiot S, Guillot B, Dehez F, Hecker A (2022) Biochemical and structural characterization of chi-class glutathione transferases: A snapshot on the glutathione transferase encoded by *sll0067* gene in the cyanobacterium *Synechocystis* sp. strain PCC 6803. *Biomolecules* 12: 1466 36291676 10.3390/biom12101466 PMC9599700

[RMuronetz2019] Muronetz VI, Melnikova AK, Barinova KV, Schmalhausen EV (2019) Inhibitors of glyceraldehyde 3-phosphate dehydrogenase and unexpected effects of its reduced activity. *Biochemistry (Mosc)* 84: 1268–1279 31760917 10.1134/S0006297919110051

[RRippka1979] Rippka R, Deruelles J, Waterbury JB, Herdman M, Stanier R (1979) Generic assignments, strain histories and properties of pure cultures of cyanobacteria. *Microbiology (Reading)* 111: 1–61

[RTakahashi2008] Takahashi H, Hihara Y, Uchimiya H (2008) Difference in metabolite levels between photoautotrophic and photomixotrophic cultures of *Synechocystis* sp. PCC 6803 examined by capillary electrophoresis electrospray ionization mass spectrometry. *J Exp Bot* 59: 3009–3018 18611912 10.1093/jxb/ern157 PMC2504344

[RThornalley1996] Thornalley PJ (1996) Pharmacology of methylglyoxal: Formation, modification of proteins and nucleic acids, and enzymatic detoxification—A role in pathogenesis and antiproliferative chemotherapy. *Gen Pharmacol* 27: 565–573 8853285 10.1016/0306-3623(95)02054-3

[d67e1295] Tötemeyer S, Booth NA, Nichols WW, Dunbar B, Booth IR (1998) From famine to feast: The role of methylglyoxal production in *Escherichia coli.* *Mol Microbiol* 27: 553–562 9489667 10.1046/j.1365-2958.1998.00700.x

[RWang2002] Wang Y, Li Y, Shi D, Shen G, Ru B, Zhang S (2002) Characteristics of mixotrophic growth of *Synechocystis* sp. in an enclosed photobioreactor. *Biotechnol Lett* 24: 1593–1597

[RYomano2009] Yomano LP, York SW, Shanmugam KT, Ingram LO (2009) Deletion of methylglyoxal synthase gene (*mgsA*) increased sugar co-metabolism in ethanol-producing *Escherichia coli.* *Biotechnol Lett* 31: 1389–1398 19458924 10.1007/s10529-009-0011-8 PMC2721133

[RYoshikawa2013] Yoshikawa K, Hirasawa T, Ogawa K, Hidaka Y, Nakajima T, Furusawa C, Shimizu H (2013) Integrated transcriptomic and metabolomic analysis of the central metabolism of *Synechocystis* sp. PCC 6803 under different trophic conditions. *Biotechnol J* 8: 571–580 23495147 10.1002/biot.201200235

[RYuan1977] Yuan PM, Gracy RW (1977) The conversion of dihydroxyacetone phosphate to methylglyoxal and inorganic phosphate by methylglyoxal synthase. *Arch Biochem Biophys* 183: 1–6 334078 10.1016/0003-9861(77)90411-8

[RZhu2001] Zhu MM, Skraly FA, Cameron DC (2001) Accumulation of methylglyoxal in anaerobically grown *Escherichia coli* and its detoxification by expression of the *Pseudomonas putida* glyoxylase I gene. *Metab Eng* 3: 218–225 11461144 10.1006/mben.2001.0186

