# Supplementary material for: The deficiency of methylglyoxal synthase promotes cell proliferation in Synechocystis sp. PCC 6803 under mixotrophic conditions
Source: Plant Biotechnol (Tokyo). 2024 Dec 25;41(4):393–9. doi: 10.5511/plantbiotechnology.24.0718a (PMC11897720; doi:10.5511/plantbiotechnology.24.0718a)
Supplement: Supplementary Data [file plantbiotechnology-41-4-24.0718a-s001.pdf]

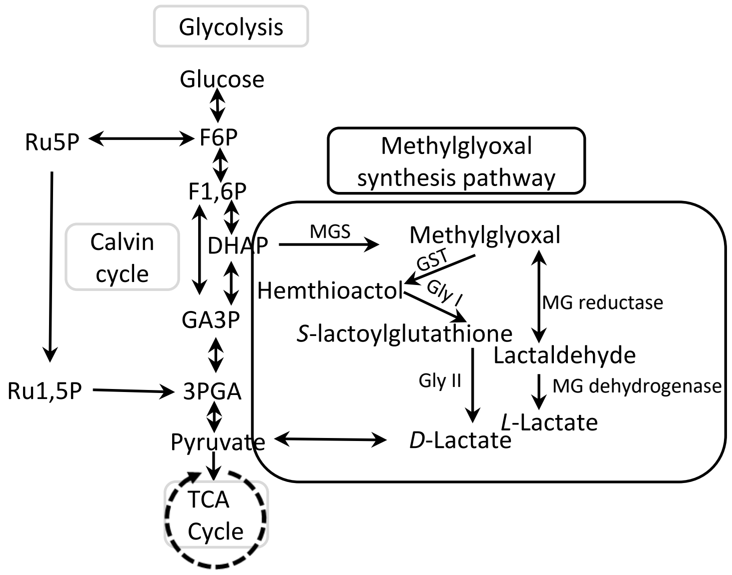


**Supplementary Figure S1.** Primary metabolism and MG synthesis pathway of cyanobacteria. MG is enzymatically synthesized from DHAP by MG synthase. GST, glutathione S-transferase; Gly I, Glyoxalase I; Gly II, Glyoxalase II, Ru5P, Ribulose 5-phosphate; Ru1,5P, Ribulose-1,5-bisphosphate.

(A)

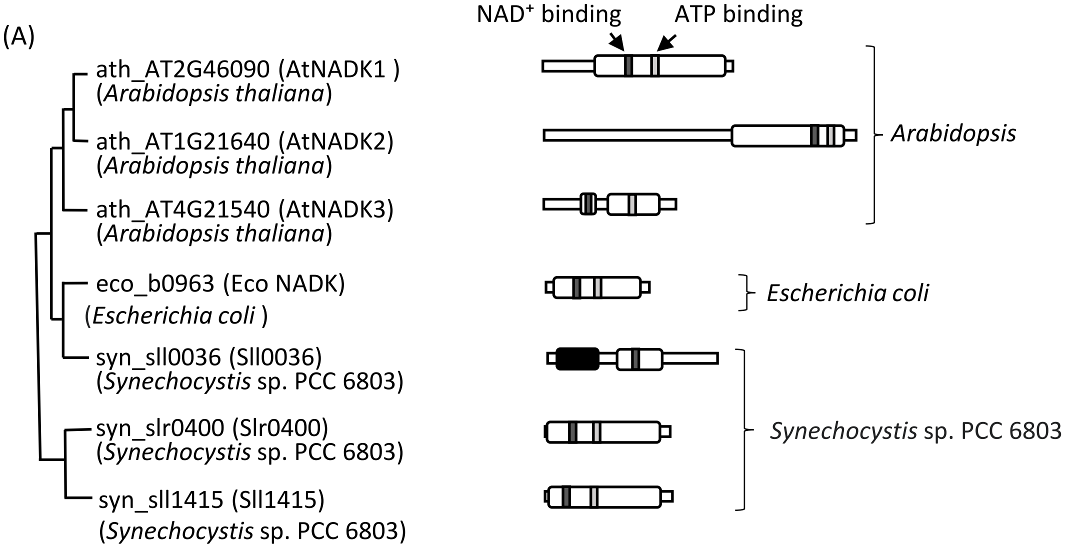


(B)

```
                        NAD+ binding site                          ATP binding site
                             GGDG                                         NE
syn_sll0036    EGDSFIIASGGDGTVSGVAAALVNTGIPLGIIPRG  ·····DRELKNNLG
ath_AT2G46090  TKVDLLITLGGDGTVLWAASMFKGPVPPIVPFSMG  ·····LNEVTIDRG
ath_AT5G23450  ERVDFVACLGGDGVILHASNLFKGAVPPVVSFNLG  ·····LNEIVVDRG
ath_AT4G21540  RDVDMVITVGGDGTLLHAS-HFIDDSVPVLGVNSD  ·····LNEIVVDRG
eco_b0963      QLADLAVVVGGDGNMLGAARTLARYDIKVIGINRG  ·····INEVVLHPG
syn_slr0400    ESMPFAIVLGGDGTVLSAFRQLAPLGIPLLTINTG  ·····LNEMVLHRE
syn_sll1415    EKIDLALVLGGDGTTLAAARHLSPEGIPILSVNVG  ·····LNEMCIKPA
```

**Supplementary Figure S2.** Comparison of genes showing homology with the NADK motif (white box) of cyanobacteria. (A) Molecular phylogenetic tree of NADK motif-containing proteins of cyanobacteria, *Arabidopsis thaliana,* and *E. coli.* (B) Amino acid alignment of $NAD^+$ binding site (GGDG) and ATP binding site (NE) of NADK motif-containing proteins.

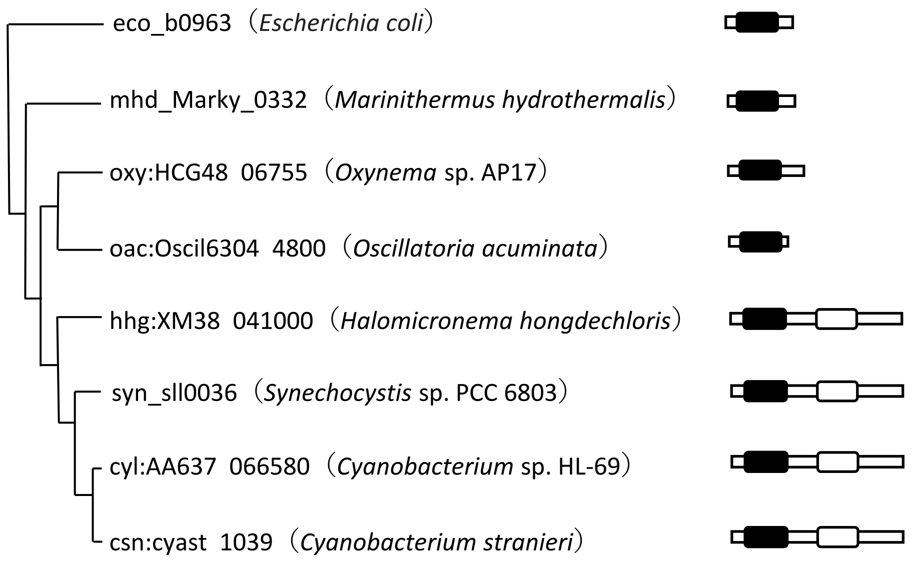


**Supplementary Figure S3.** Molecular phylogenetic tree of genes with MG synthase motifs. MG synthase exists only in prokaryotes; one group has only MG synthase (black box) and the other group has both MGS and NADK (white box) motifs.
